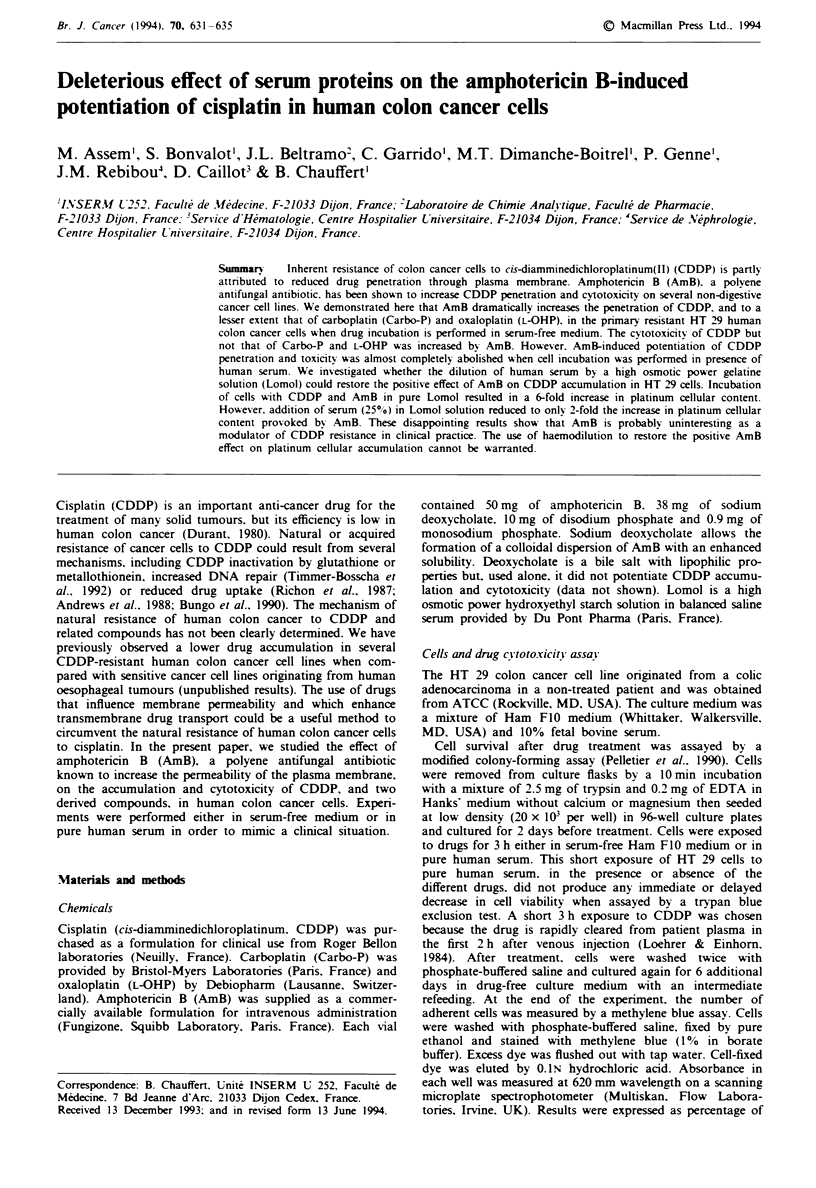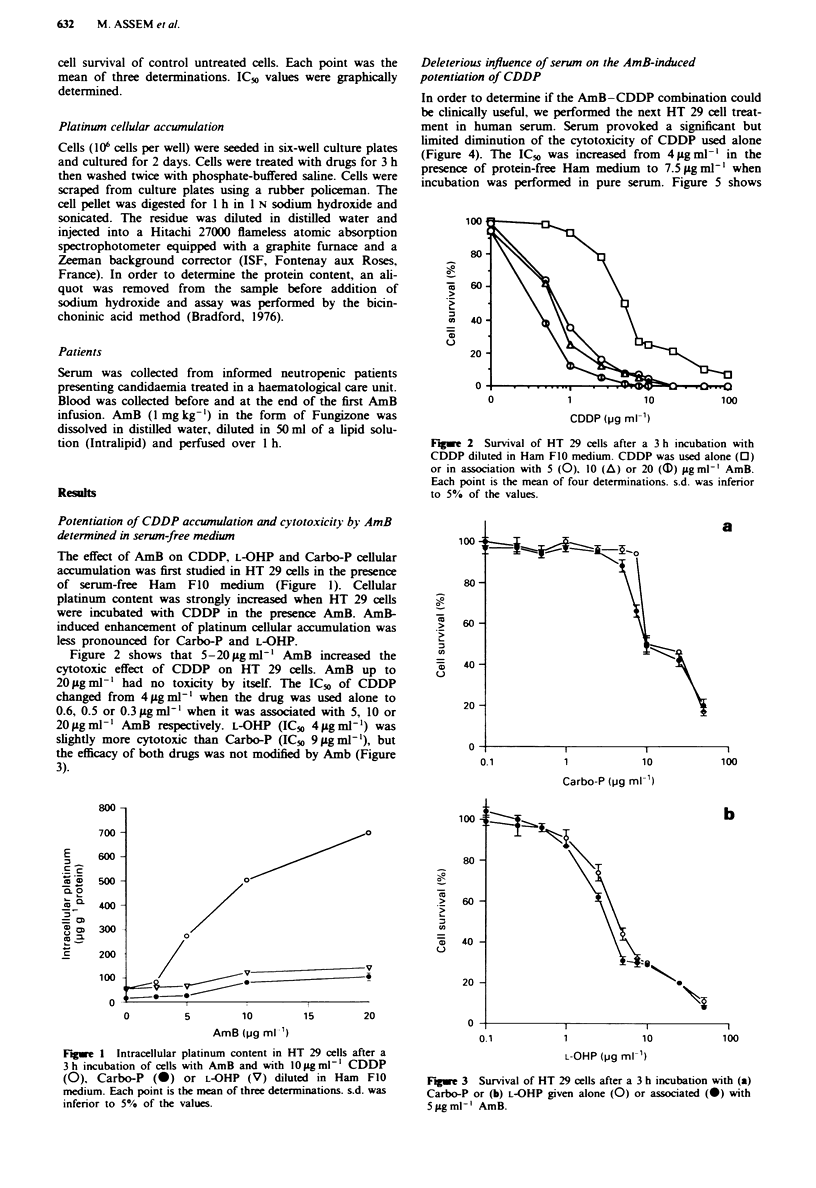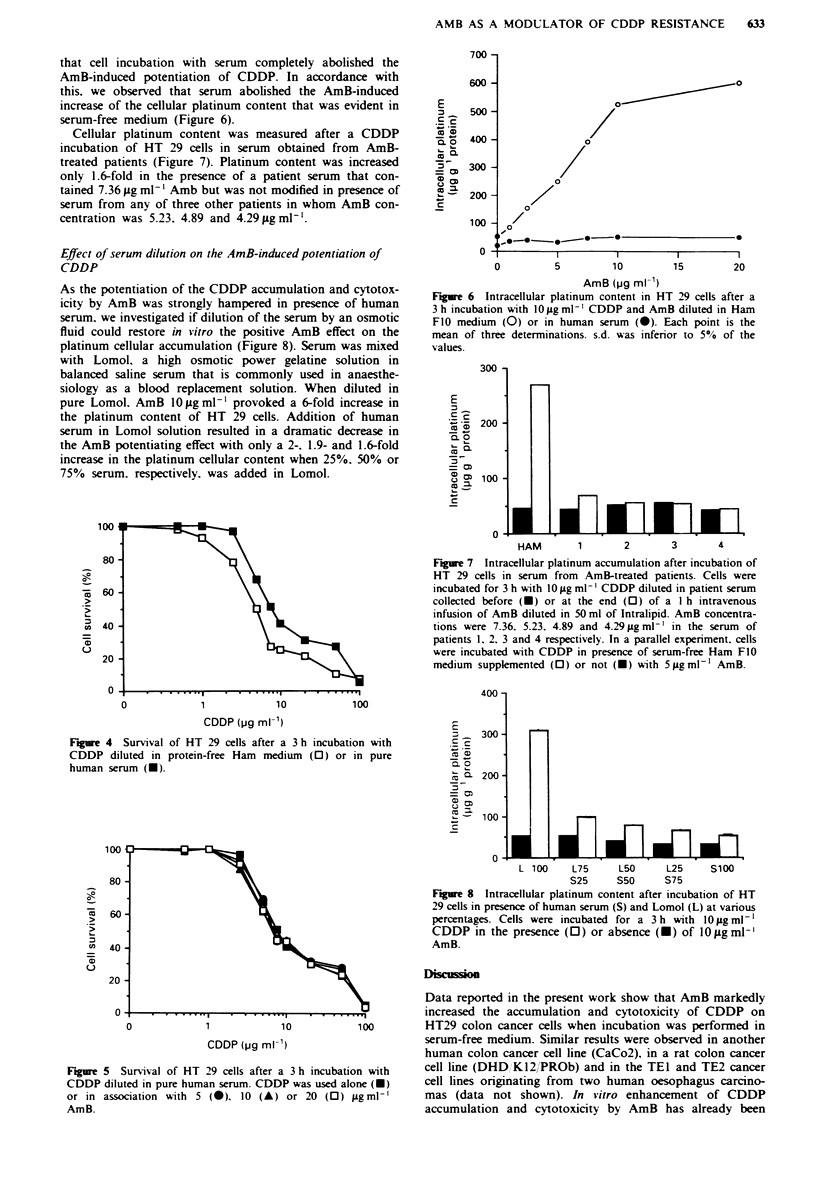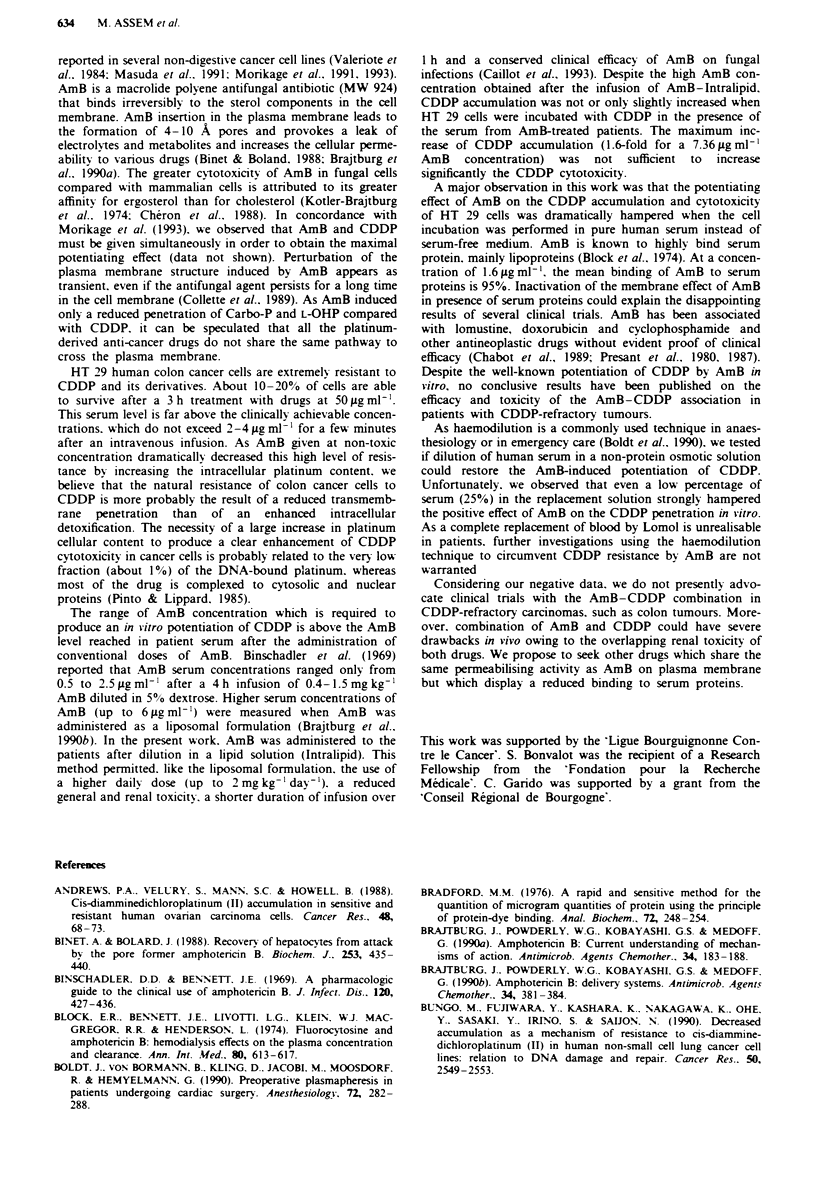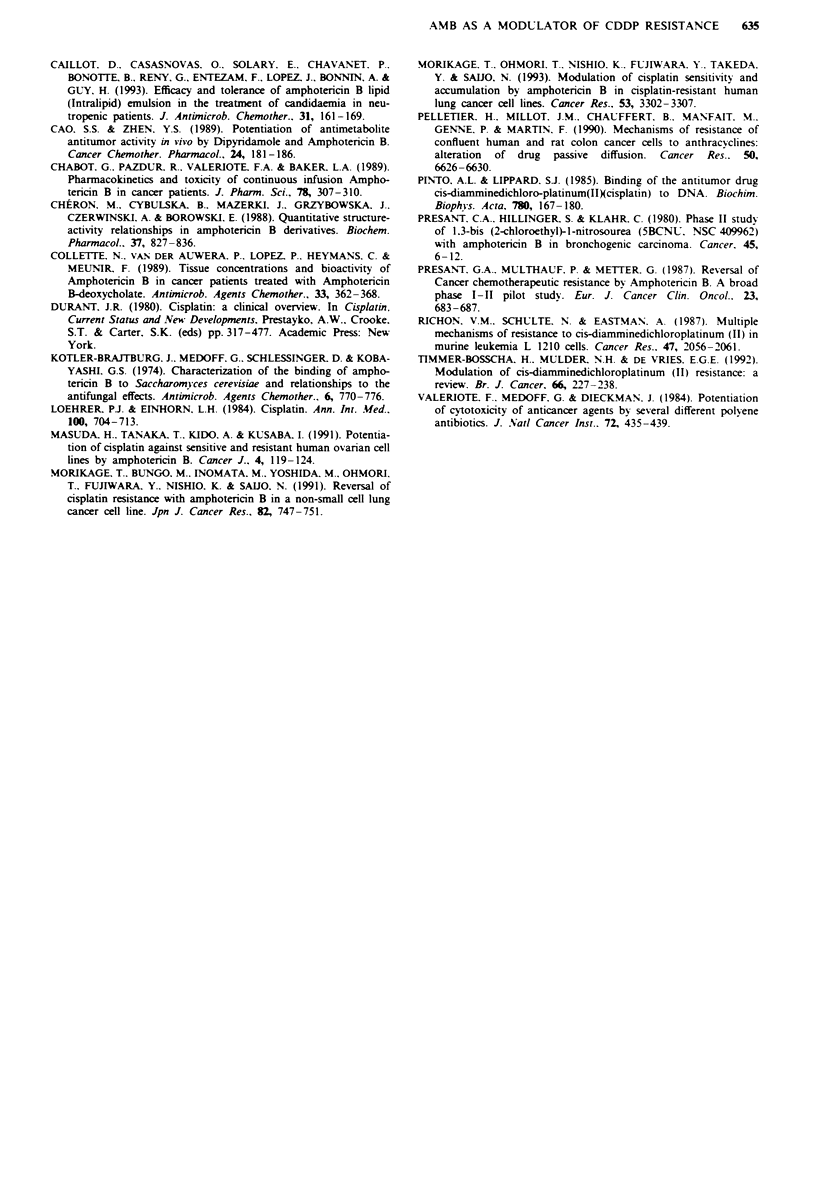# Deleterious effect of serum proteins on the amphotericin B-induced potentiation of cisplatin in human colon cancer cells.

**DOI:** 10.1038/bjc.1994.362

**Published:** 1994-10

**Authors:** M. Assem, S. Bonvalot, J. L. Beltramo, C. Garrido, M. T. Dimanche-Boitrel, P. Genne, J. M. Rebibou, D. Caillot, B. Chauffert

**Affiliations:** INSERM U252, Faculté de Médecine, Dijon, France.

## Abstract

Inherent resistance of colon cancer cells to cis-diamminedichloroplatinum(II) (CDDP) is partly attributed to reduced drug penetration through plasma membrane. Amphotericin B (AmB), a polyene antifungal antibiotic, has been shown to increase CDDP penetration and cytotoxicity on several non-digestive cancer cell lines. We demonstrated here that AmB dramatically increases the penetration of CDDP, and to a lesser extent that of carboplatin (Carbo-P) and oxaloplatin (L-OHP), in the primary resistant HT 29 human colon cancer cells when drug incubation is performed in serum-free medium. The cytotoxicity of CDDP but not that of Carbo-P and L-OHP was increased by AmB. However, AmB-induced potentiation of CDDP penetration and toxicity was almost completely abolished when cell incubation was performed in presence of human serum. We investigated whether the dilution of human serum by a high osmotic power gelatine solution (Lomol) could restore the positive effect of AmB on CDDP accumulation in HT 29 cells. Incubation of cells with CDDP and AmB in pure Lomol resulted in a 6-fold increase in platinum cellular content. However, addition of serum (25%) in Lomol solution reduced to only 2-fold the increase in platinum cellular content provoked by AmB. These disappointing results show that AmB is probably uninteresting as a modulator of CDDP resistance in clinical practice. The use of haemodilution to restore the positive AmB effect on platinum cellular accumulation cannot be warranted.


Br. J. Cancer (1994), 70, 631-635 © Macmillan Press Ltd., 1994

### Deleterious effect of serum proteins on the amphotericin B-induced potentiation of cisplatin in human colon cancer cells

M. Assem[1], S. Bonvalot[1], J.L. Beltramo[2], C. Garrido[1], M.T. Dimanche-Boitrel[1], P. Genne[1], J.M. Rebibou[4], D. Caillot[3] & B. Chauffert[1]

[1]INSERM U252, Faculté de Médecine, F-21033 Dijon, France; [2]Laboratoire de Chimie Analytique, Faculté de Pharmacie, F-21033 Dijon, France; [3]Service d'Hématologie, Centre Hospitalier Universitaire, F-21034 Dijon, France; [4]Service de Néphrologie, Centre Hospitalier Universitaire, F-21034 Dijon, France.

**Summary** Inherent resistance of colon cancer cells to *cis*-diamminedichloroplatinum(II) (CDDP) is partly attributed to reduced drug penetration through plasma membrane. Amphotericin B (AmB), a polyene antifungal antibiotic, has been shown to increase CDDP penetration and cytotoxicity on several non-digestive cancer cell lines. We demonstrated here that AmB dramatically increases the penetration of CDDP, and to a lesser extent that of carboplatin (Carbo-P) and oxaloplatin (L-OHP), in the primary resistant HT 29 human colon cancer cells when drug incubation is performed in serum-free medium. The cytotoxicity of CDDP but not that of Carbo-P and L-OHP was increased by AmB. However, AmB-induced potentiation of CDDP penetration and toxicity was almost completely abolished when cell incubation was performed in presence of human serum. We investigated whether the dilution of human serum by a high osmotic power gelatine solution (Lomol) could restore the positive effect of AmB on CDDP accumulation in HT 29 cells. Incubation of cells with CDDP and AmB in pure Lomol resulted in a 6-fold increase in platinum cellular content. However, addition of serum (25%) in Lomol solution reduced to only 2-fold the increase in platinum cellular content provoked by AmB. These disappointing results show that AmB is probably uninteresting as a modulator of CDDP resistance in clinical practice. The use of haemodilution to restore the positive AmB effect on platinum cellular accumulation cannot be warranted.

Cisplatin (CDDP) is an important anti-cancer drug for the treatment of many solid tumours, but its efficiency is low in human colon cancer (Durant, 1980). Natural or acquired resistance of cancer cells to CDDP could result from several mechanisms, including CDDP inactivation by glutathione or metallothionein, increased DNA repair (Timmer-Bosscha *et al*., 1992) or reduced drug uptake (Richon *et al*., 1987; Andrews *et al*., 1988; Bungo *et al*., 1990). The mechanism of natural resistance of human colon cancer to CDDP and related compounds has not been clearly determined. We have previously observed a lower drug accumulation in several CDDP-resistant human colon cancer cell lines when compared with sensitive cancer cell lines originating from human oesophageal tumours (unpublished results). The use of drugs that influence membrane permeability and which enhance transmembrane drug transport could be a useful method to circumvent the natural resistance of human colon cancer cells to cisplatin. In the present paper, we studied the effect of amphotericin B (AmB), a polyene antifungal antibiotic known to increase the permeability of the plasma membrane, on the accumulation and cytotoxicity of CDDP, and two derived compounds, in human colon cancer cells. Experiments were performed either in serum-free medium or in pure human serum in order to mimic a clinical situation.

### Materials and methods

### Chemicals

Cisplatin (*cis*-diamminedichloroplatinum, CDDP) was purchased as a formulation for clinical use from Roger Bellon laboratories (Neuilly, France). Carboplatin (Carbo-P) was provided by Bristol-Myers Laboratories (Paris, France) and oxaloplatin (L-OHP) by Debiopharm (Lausanne, Switzerland). Amphotericin B (AmB) was supplied as a commercially available formulation for intravenous administration (Fungizone, Squibb Laboratory, Paris, France). Each vial contained 50 mg of amphotericin B, 38 mg of sodium deoxycholate, 10 mg of disodium phosphate and 0.9 mg of monosodium phosphate. Sodium deoxycholate allows the formation of a colloidal dispersion of AmB with an enhanced solubility. Deoxycholate is a bile salt with lipophilic properties but, used alone, it did not potentiate CDDP accumulation and cytotoxicity (data not shown). Lomol is a high osmotic power hydroxyethyl starch solution in balanced saline serum provided by Du Pont Pharma (Paris, France).

### Cells and drug cytotoxicity assay

The HT 29 colon cancer cell line originated from a colic adenocarcinoma in a non-treated patient and was obtained from ATCC (Rockville, MD, USA). The culture medium was a mixture of Ham F10 medium (Whittaker, Walkersville, MD, USA) and 10% fetal bovine serum.

Cell survival after drug treatment was assayed by a modified colony-forming assay (Pelletier *et al*., 1990). Cells were removed from culture flasks by a 10 min incubation with a mixture of 2.5 mg of trypsin and 0.2 mg of EDTA in Hanks' medium without calcium or magnesium then seeded at low density ($20 \times 10^3$ per well) in 96-well culture plates and cultured for 2 days before treatment. Cells were exposed to drugs for 3 h either in serum-free Ham F10 medium or in pure human serum. This short exposure of HT 29 cells to pure human serum, in the presence or absence of the different drugs, did not produce any immediate or delayed decrease in cell viability when assayed by a trypan blue exclusion test. A short 3 h exposure to CDDP was chosen because the drug is rapidly cleared from patient plasma in the first 2 h after venous injection (Loehrer & Einhorn, 1984). After treatment, cells were washed twice with phosphate-buffered saline and cultured again for 6 additional days in drug-free culture medium with an intermediate refeeding. At the end of the experiment, the number of adherent cells was measured by a methylene blue assay. Cells were washed with phosphate-buffered saline, fixed by pure ethanol and stained with methylene blue (1% in borate buffer). Excess dye was flushed out with tap water. Cell-fixed dye was eluted by 0.1N hydrochloric acid. Absorbance in each well was measured at 620 mm wavelength on a scanning microplate spectrophotometer (Multiskan, Flow Laboratories, Irvine, UK). Results were expressed as percentage of

Correspondence: B. Chauffert, Unité INSERM U 252, Faculté de Médecine, 7 Bd Jeanne d'Arc, 21033 Dijon Cedex, France.
Received 13 December 1993; and in revised form 13 June 1994.

cell survival of control untreated cells. Each point was the mean of three determinations. $IC_{50}$ values were graphically determined.

*Platinum cellular accumulation*

Cells ($10^6$ cells per well) were seeded in six-well culture plates and cultured for 2 days. Cells were treated with drugs for 3 h then washed twice with phosphate-buffered saline. Cells were scraped from culture plates using a rubber policeman. The cell pellet was digested for 1 h in 1 N sodium hydroxide and sonicated. The residue was diluted in distilled water and injected into a Hitachi 27000 flameless atomic absorption spectrophotometer equipped with a graphite furnace and a Zeeman background corrector (ISF, Fontenay aux Roses, France). In order to determine the protein content, an aliquot was removed from the sample before addition of sodium hydroxide and assay was performed by the bicinchoninic acid method (Bradford, 1976).

*Patients*

Serum was collected from informed neutropenic patients presenting candidaemia treated in a haematological care unit. Blood was collected before and at the end of the first AmB infusion. AmB (1 mg $kg^{-1}$) in the form of Fungizone was dissolved in distilled water, diluted in 50 ml of a lipid solution (Intralipid) and perfused over 1 h.

### Results

*Potentiation of CDDP accumulation and cytotoxicity by AmB determined in serum-free medium*

The effect of AmB on CDDP, L-OHP and Carbo-P cellular accumulation was first studied in HT 29 cells in the presence of serum-free Ham F10 medium (Figure 1). Cellular platinum content was strongly increased when HT 29 cells were incubated with CDDP in the presence AmB. AmB-induced enhancement of platinum cellular accumulation was less pronounced for Carbo-P and L-OHP.

Figure 2 shows that 5–20 μg $ml^{-1}$ AmB increased the cytotoxic effect of CDDP on HT 29 cells. AmB up to 20 μg $ml^{-1}$ had no toxicity by itself. The $IC_{50}$ of CDDP changed from 4 μg $ml^{-1}$ when the drug was used alone to 0.6, 0.5 or 0.3 μg $ml^{-1}$ when it was associated with 5, 10 or 20 μg $ml^{-1}$ AmB respectively. L-OHP ($IC_{50}$ 4 μg $ml^{-1}$) was slightly more cytotoxic than Carbo-P ($IC_{50}$ 9 μg $ml^{-1}$), but the efficacy of both drugs was not modified by Amb (Figure 3).

**Figure 1** Intracellular platinum content in HT 29 cells after a 3 h incubation of cells with AmB and with 10 μg $ml^{-1}$ CDDP (○), Carbo-P (●) or L-OHP (▽) diluted in Ham F10 medium. Each point is the mean of three determinations. s.d. was inferior to 5% of the values.

*Deleterious influence of serum on the AmB-induced potentiation of CDDP*

In order to determine if the AmB–CDDP combination could be clinically useful, we performed the next HT 29 cell treatment in human serum. Serum provoked a significant but limited diminution of the cytotoxicity of CDDP used alone (Figure 4). The $IC_{50}$ was increased from 4 μg $ml^{-1}$ in the presence of protein-free Ham medium to 7.5 μg $ml^{-1}$ when incubation was performed in pure serum. Figure 5 shows

**Figure 2** Survival of HT 29 cells after a 3 h incubation with CDDP diluted in Ham F10 medium. CDDP was used alone (□) or in association with 5 (○), 10 (△) or 20 (⦶) μg $ml^{-1}$ AmB. Each point is the mean of four determinations. s.d. was inferior to 5% of the values.

**Figure 3** Survival of HT 29 cells after a 3 h incubation with (**a**) Carbo-P or (**b**) L-OHP given alone (○) or associated (●) with 5 μg $ml^{-1}$ AmB.

that cell incubation with serum completely abolished the AmB-induced potentiation of CDDP. In accordance with this, we observed that serum abolished the AmB-induced increase of the cellular platinum content that was evident in serum-free medium (Figure 6).

Cellular platinum content was measured after a CDDP incubation of HT 29 cells in serum obtained from AmB-treated patients (Figure 7). Platinum content was increased only 1.6-fold in the presence of a patient serum that contained 7.36 $\mu g\ ml^{-1}$ Amb but was not modified in presence of serum from any of three other patients in whom AmB concentration was 5.23, 4.89 and 4.29 $\mu g\ ml^{-1}$.

### *Effect of serum dilution on the AmB-induced potentiation of CDDP*

As the potentiation of the CDDP accumulation and cytotoxicity by AmB was strongly hampered in presence of human serum, we investigated if dilution of the serum by an osmotic fluid could restore *in vitro* the positive AmB effect on the platinum cellular accumulation (Figure 8). Serum was mixed with Lomol, a high osmotic power gelatine solution in balanced saline serum that is commonly used in anaesthesiology as a blood replacement solution. When diluted in pure Lomol, AmB 10 $\mu g\ ml^{-1}$ provoked a 6-fold increase in the platinum content of HT 29 cells. Addition of human serum in Lomol solution resulted in a dramatic decrease in the AmB potentiating effect with only a 2-, 1.9- and 1.6-fold increase in the platinum cellular content when 25%, 50% or 75% serum, respectively, was added in Lomol.

**Figure 4** Survival of HT 29 cells after a 3 h incubation with CDDP diluted in protein-free Ham medium (□) or in pure human serum (■).

**Figure 5** Survival of HT 29 cells after a 3 h incubation with CDDP diluted in pure human serum. CDDP was used alone (■) or in association with 5 (●), 10 (▲) or 20 (□) $\mu g\ ml^{-1}$ AmB.

**Figure 6** Intracellular platinum content in HT 29 cells after a 3 h incubation with 10 $\mu g\ ml^{-1}$ CDDP and AmB diluted in Ham F10 medium (○) or in human serum (●). Each point is the mean of three determinations. s.d. was inferior to 5% of the values.

**Figure 7** Intracellular platinum accumulation after incubation of HT 29 cells in serum from AmB-treated patients. Cells were incubated for 3 h with 10 $\mu g\ ml^{-1}$ CDDP diluted in patient serum collected before (■) or at the end (□) of a 1 h intravenous infusion of AmB diluted in 50 ml of Intralipid. AmB concentrations were 7.36, 5.23, 4.89 and 4.29 $\mu g\ ml^{-1}$ in the serum of patients 1, 2, 3 and 4 respectively. In a parallel experiment, cells were incubated with CDDP in presence of serum-free Ham F10 medium supplemented (□) or not (■) with 5 $\mu g\ ml^{-1}$ AmB.

**Figure 8** Intracellular platinum content after incubation of HT 29 cells in presence of human serum (S) and Lomol (L) at various percentages. Cells were incubated for a 3 h with 10 $\mu g\ ml^{-1}$ CDDP in the presence (□) or absence (■) of 10 $\mu g\ ml^{-1}$ AmB.

### Discussion

Data reported in the present work show that AmB markedly increased the accumulation and cytotoxicity of CDDP on HT29 colon cancer cells when incubation was performed in serum-free medium. Similar results were observed in another human colon cancer cell line (CaCo2), in a rat colon cancer cell line (DHD/K12/PROb) and in the TE1 and TE2 cancer cell lines originating from two human oesophagus carcinomas (data not shown). *In vitro* enhancement of CDDP accumulation and cytotoxicity by AmB has already been reported in several non-digestive cancer cell lines (Valeriote et al., 1984; Masuda et al., 1991; Morikage et al., 1991, 1993). AmB is a macrolide polyene antifungal antibiotic (MW 924) that binds irreversibly to the sterol components in the cell membrane. AmB insertion in the plasma membrane leads to the formation of 4–10 Å pores and provokes a leak of electrolytes and metabolites and increases the cellular permeability to various drugs (Binet & Boland, 1988; Brajtburg et al., 1990a). The greater cytotoxicity of AmB in fungal cells compared with mammalian cells is attributed to its greater affinity for ergosterol than for cholesterol (Kotler-Brajtburg et al., 1974; Chéron et al., 1988). In concordance with Morikage et al. (1993), we observed that AmB and CDDP must be given simultaneously in order to obtain the maximal potentiating effect (data not shown). Perturbation of the plasma membrane structure induced by AmB appears as transient, even if the antifungal agent persists for a long time in the cell membrane (Collette et al., 1989). As AmB induced only a reduced penetration of Carbo-P and L-OHP compared with CDDP, it can be speculated that all the platinum-derived anti-cancer drugs do not share the same pathway to cross the plasma membrane.

HT 29 human colon cancer cells are extremely resistant to CDDP and its derivatives. About 10–20% of cells are able to survive after a 3 h treatment with drugs at 50 $\mu g\ ml^{-1}$. This serum level is far above the clinically achievable concentrations, which do not exceed 2–4 $\mu g\ ml^{-1}$ for a few minutes after an intravenous infusion. As AmB given at non-toxic concentration dramatically decreased this high level of resistance by increasing the intracellular platinum content, we believe that the natural resistance of colon cancer cells to CDDP is more probably the result of a reduced transmembrane penetration than of an enhanced intracellular detoxification. The necessity of a large increase in platinum cellular content to produce a clear enhancement of CDDP cytotoxicity in cancer cells is probably related to the very low fraction (about 1%) of the DNA-bound platinum, whereas most of the drug is complexed to cytosolic and nuclear proteins (Pinto & Lippard, 1985).

The range of AmB concentration which is required to produce an in vitro potentiation of CDDP is above the AmB level reached in patient serum after the administration of conventional doses of AmB. Binschadler et al. (1969) reported that AmB serum concentrations ranged only from 0.5 to 2.5 $\mu g\ ml^{-1}$ after a 4 h infusion of 0.4–1.5 $mg\ kg^{-1}$ AmB diluted in 5% dextrose. Higher serum concentrations of AmB (up to 6 $\mu g\ ml^{-1}$) were measured when AmB was administered as a liposomal formulation (Brajtburg et al., 1990b). In the present work, AmB was administered to the patients after dilution in a lipid solution (Intralipid). This method permitted, like the liposomal formulation, the use of a higher daily dose (up to 2 $mg\ kg^{-1}\ day^{-1}$), a reduced general and renal toxicity, a shorter duration of infusion over 1 h and a conserved clinical efficacy of AmB on fungal infections (Caillot et al., 1993). Despite the high AmB concentration obtained after the infusion of AmB–Intralipid, CDDP accumulation was not or only slightly increased when HT 29 cells were incubated with CDDP in the presence of the serum from AmB-treated patients. The maximum increase of CDDP accumulation (1.6-fold for a 7.36 $\mu g\ ml^{-1}$ AmB concentration) was not sufficient to increase significantly the CDDP cytotoxicity.

A major observation in this work was that the potentiating effect of AmB on the CDDP accumulation and cytotoxicity of HT 29 cells was dramatically hampered when the cell incubation was performed in pure human serum instead of serum-free medium. AmB is known to highly bind serum protein, mainly lipoproteins (Block et al., 1974). At a concentration of 1.6 $\mu g\ ml^{-1}$, the mean binding of AmB to serum proteins is 95%. Inactivation of the membrane effect of AmB in presence of serum proteins could explain the disappointing results of several clinical trials. AmB has been associated with lomustine, doxorubicin and cyclophosphamide and other antineoplastic drugs without evident proof of clinical efficacy (Chabot et al., 1989; Presant et al., 1980, 1987). Despite the well-known potentiation of CDDP by AmB in vitro, no conclusive results have been published on the efficacy and toxicity of the AmB–CDDP association in patients with CDDP-refractory tumours.

As haemodilution is a commonly used technique in anaesthesiology or in emergency care (Boldt et al., 1990), we tested if dilution of human serum in a non-protein osmotic solution could restore the AmB-induced potentiation of CDDP. Unfortunately, we observed that even a low percentage of serum (25%) in the replacement solution strongly hampered the positive effect of AmB on the CDDP penetration in vitro. As a complete replacement of blood by Lomol is unrealisable in patients, further investigations using the haemodilution technique to circumvent CDDP resistance by AmB are not warranted

Considering our negative data, we do not presently advocate clinical trials with the AmB–CDDP combination in CDDP-refractory carcinomas, such as colon tumours. Moreover, combination of AmB and CDDP could have severe drawbacks in vivo owing to the overlapping renal toxicity of both drugs. We propose to seek other drugs which share the same permeabilising activity as AmB on plasma membrane but which display a reduced binding to serum proteins.

This work was supported by the 'Ligue Bourguignonne Contre le Cancer'. S. Bonvalot was the recipient of a Research Fellowship from the 'Fondation pour la Recherche Médicale'. C. Garido was supported by a grant from the 'Conseil Régional de Bourgogne'.

### References

ANDREWS, P.A., VELURY, S., MANN, S.C. & HOWELL, B. (1988). Cis-diamminedichloroplatinum (II) accumulation in sensitive and resistant human ovarian carcinoma cells. Cancer Res., **48**, 68–73.

BINET, A. & BOLARD, J. (1988). Recovery of hepatocytes from attack by the pore former amphotericin B. Biochem. J., **253**, 435–440.

BINSCHADLER, D.D. & BENNETT, J.E. (1969). A pharmacologic guide to the clinical use of amphotericin B. J. Infect. Dis., **120**, 427–436.

BLOCK, E.R., BENNETT, J.E., LIVOTTI, L.G., KLEIN, W.J. MACGREGOR, R.R. & HENDERSON, L. (1974). Fluorocytosine and amphotericin B: hemodialysis effects on the plasma concentration and clearance. Ann. Int. Med., **80**, 613–617.

BOLDT, J., VON BORMANN, B., KLING, D., JACOBI, M., MOOSDORF, R. & HEMYELMANN, G. (1990). Preoperative plasmapheresis in patients undergoing cardiac surgery. Anesthesiology, **72**, 282–288.

BRADFORD, M.M. (1976). A rapid and sensitive method for the quantition of microgram quantities of protein using the principle of protein-dye binding. Anal. Biochem., **72**, 248–254.

BRAJTBURG, J., POWDERLY, W.G., KOBAYASHI, G.S. & MEDOFF, G. (1990a). Amphotericin B: Current understanding of mechanisms of action. Antimicrob. Agents Chemother., **34**, 183–188.

BRAJTBURG, J., POWDERLY, W.G., KOBAYASHI, G.S. & MEDOFF, G. (1990b). Amphotericin B: delivery systems. Antimicrob. Agents Chemother., **34**, 381–384.

BUNGO, M., FUJIWARA, Y., KASHARA, K., NAKAGAWA, K., OHE, Y., SASAKI, Y., IRINO, S. & SAIJON, N. (1990). Decreased accumulation as a mechanism of resistance to cis-diamminedichloroplatinum (II) in human non-small cell lung cancer cell lines: relation to DNA damage and repair. Cancer Res., **50**, 2549–2553.

CAILLOT, D., CASASNOVAS, O., SOLARY, E., CHAVANET, P., BONOTTE, B., RENY, G., ENTEZAM, F., LOPEZ, J., BONNIN, A. & GUY, H. (1993). Efficacy and tolerance of amphotericin B lipid (Intralipid) emulsion in the treatment of candidaemia in neutropenic patients. *J. Antimicrob. Chemother.*, **31**, 161–169.

CAO, S.S. & ZHEN, Y.S. (1989). Potentiation of antimetabolite antitumor activity *in vivo* by Dipyridamole and Amphotericin B. *Cancer Chemother. Pharmacol.*, **24**, 181–186.

CHABOT, G., PAZDUR, R., VALERIOTE, F.A. & BAKER, L.A. (1989). Pharmacokinetics and toxicity of continuous infusion Amphotericin B in cancer patients. *J. Pharm. Sci.*, **78**, 307–310.

CHÉRON, M., CYBULSKA, B., MAZERKI, J., GRZYBOWSKA, J., CZERWINSKI, A. & BOROWSKI, E. (1988). Quantitative structure-activity relationships in amphotericin B derivatives. *Biochem. Pharmacol.*, **37**, 827–836.

COLLETTE, N., VAN DER AUWERA, P., LOPEZ, P., HEYMANS, C. & MEUNIR, F. (1989). Tissue concentrations and bioactivity of Amphotericin B in cancer patients treated with Amphotericin B-deoxycholate. *Antimicrob. Agents Chemother.*, **33**, 362–368.

DURANT, J.R. (1980). Cisplatin: a clinical overview. In *Cisplatin, Current Status and New Developments*, Prestayko, A.W., Crooke, S.T. & Carter, S.K. (eds) pp. 317–477. Academic Press: New York.

KOTLER-BRAJTBURG, J., MEDOFF, G., SCHLESSINGER, D. & KOBAYASHI, G.S. (1974). Characterization of the binding of amphotericin B to *Saccharomyces cerevisiae* and relationships to the antifungal effects. *Antimicrob. Agents Chemother.*, **6**, 770–776.

LOEHRER, P.J. & EINHORN, L.H. (1984). Cisplatin. *Ann. Int. Med.*, **100**, 704–713.

MASUDA, H., TANAKA, T., KIDO, A. & KUSABA, I. (1991). Potentiation of cisplatin against sensitive and resistant human ovarian cell lines by amphotericin B. *Cancer J.*, **4**, 119–124.

MORIKAGE, T., BUNGO, M., INOMATA, M., YOSHIDA, M., OHMORI, T., FUJIWARA, Y., NISHIO, K. & SAIJO, N. (1991). Reversal of cisplatin resistance with amphotericin B in a non-small cell lung cancer cell line. *Jpn J. Cancer Res.*, **82**, 747–751.

MORIKAGE, T., OHMORI, T., NISHIO, K., FUJIWARA, Y., TAKEDA, Y. & SAIJO, N. (1993). Modulation of cisplatin sensitivity and accumulation by amphotericin B in cisplatin-resistant human lung cancer cell lines. *Cancer Res.*, **53**, 3302–3307.

PELLETIER, H., MILLOT, J.M., CHAUFFERT, B., MANFAIT, M., GENNE, P. & MARTIN, F. (1990). Mechanisms of resistance of confluent human and rat colon cancer cells to anthracyclines: alteration of drug passive diffusion. *Cancer Res.*, **50**, 6626–6630.

PINTO, A.L. & LIPPARD, S.J. (1985). Binding of the antitumor drug cis-diamminedichloro-platinum(II)(cisplatin) to DNA. *Biochim. Biophys. Acta*, **780**, 167–180.

PRESANT, C.A., HILLINGER, S. & KLAHR, C. (1980). Phase II study of 1,3-bis (2-chloroethyl)-1-nitrosourea (5BCNU, NSC 409962) with amphotericin B in bronchogenic carcinoma. *Cancer*, **45**, 6–12.

PRESANT, G.A., MULTHAUF, P. & METTER, G. (1987). Reversal of Cancer chemotherapeutic resistance by Amphotericin B. A broad phase I–II pilot study. *Eur. J. Cancer Clin. Oncol.*, **23**, 683–687.

RICHON, V.M., SCHULTE, N. & EASTMAN, A. (1987). Multiple mechanisms of resistance to cis-diamminedichloroplatinum (II) in murine leukemia L 1210 cells. *Cancer Res.*, **47**, 2056–2061.

TIMMER-BOSSCHA, H., MULDER, N.H. & DE VRIES, E.G.E. (1992). Modulation of cis-diamminedichloroplatinum (II) resistance: a review. *Br. J. Cancer*, **66**, 227–238.

VALERIOTE, F., MEDOFF, G. & DIECKMAN, J. (1984). Potentiation of cytotoxicity of anticancer agents by several different polyene antibiotics. *J. Natl Cancer Inst.*, **72**, 435–439.